# Draft Genome Sequence of *Acinetobacter baumannii* IITR88, a Bacterium Degrading Indoles and Other Aromatic Compounds

**DOI:** 10.1128/genomeA.00065-16

**Published:** 2016-03-03

**Authors:** Raj Kumar Regar, Vivek Kumar Gaur, Gayatri Mishra, Sudhir Jadhao, Mohan Kamthan, Natesan Manickam

**Affiliations:** a Environmental Biotechnology Laboratory, Environmental Toxicology Group, CSIR Indian Institute of Toxicology Research (IITR), Vishvigyan Bhawan, Lucknow, India; b Bionivid Technology Pvt. Ltd., Bengaluru, Karnataka, India

## Abstract

Here, we report the 4.16-Mb draft genome sequence of an indole-degrading bacterium, *Acinetobacter baumannii* IITR88, isolated from the Bhagirathi river in India. A total of 4,069 coding regions (CDSs), 3 rRNAs, and 52 tRNAs were predicted. Genes for the degradation of indoles, phenylacetaldehyde, anthranilate, and several other aromatic compounds were present.

## GENOME ANNOUNCEMENT

The genome of *Acinetobacter baumannii* IITR88, a Gram-negative and aerobic bacterium, was sequenced here. Illumina platform paired-end technology produced a total of 33,357,632 paired-end reads of 151 bp. We used the NGS QC toolkit version 2.3 (1) to filter high-quality (HQ) data (cutoff read length for HQ, 70%; cutoff quality score, 20) for genome assembly. A total of 29,571,406 reads were used for the assembly with Velvet 1.2.08 (2) (at a k-mer length of 69). Based on the paired-end directional information, the genome was assembled into 43 scaffolds, with an *N*_50_ of 270,137 bp and average scaffold length of 96,871 bp using the SSPACE version 3.0 (3) scaffolder. The genome was assembled, resulting in a genome size of ~4.16 Mb, with a G+C content of 38.97%.

The input sequences assembled into 43 scaffolds and were anchored onto the nearest reference genome of *A. baumannii* AB5256 using CONTIGuator (4). The final genome draft of strain IITR88 consists of a pseudochromosome and unaligned scaffolds of length ≥218 bp. The final genome draft of strain IITR88 consists of 22 scaffolds, with an average length of 181,193.87 bp and *N*_50_ size of 3,990,690 bp, constituting a genome size of 4,167,459 bp.

The draft genome (22 scaffolds), comprising 4,167,459 nucleotides, was annotated with the help of the Rapid Annotations using Subsystems Technology (RAST) version 2.0 (5), Prodigal version 2.6.2 (6), and RNAmmer 1.2 servers (7). Three types of rRNAs and possible tRNAs were identified, indicating a high degree of completeness in the genome assembly. A total of 4,077 genes predicted from Prodigal, 4,069 predicted coding regions (CDSs), and 3 rRNAs (16S rRNA, 28S rRNA, and 5S rRNA) were predicted. ARAGORN version 1.2.36 (8) predicted 52 tRNAs. No plasmid was identified when it was analyzed using Webcutter (version 2.0) and PlasmidFinder (version 1.3) (9), and 3 prophages were identified using the PHAST server (10). Taxonomic identification using EzTaxon (11) and MEGA6 revealed that *Acinetobacter venetianus* and *Acinetobacter junii* are putative species, and *A. baumannii* is the closest homolog of the assembled genome.

RAST annotation shows that *A. baumannii* strains AB5256 (score, 539), AB4857 (score, 516), and TYTH-1 (score, 500) are the closest neighbors of *A. baumannii* IITR88. RAST annotation also shows that strain IITR88 contains the 3,072 characterized proteins, 1,046 hypothetical/putative proteins, and 1,146 proteins with Pathway Annotation. Strain IITR88 has genes coding for the enzymes indole-3-glycerol phosphate synthase (EC 4.1.1.48), catechol 1,2-dioxygenase (EC 1.13.11.1), benzoate 1,2-dioxygenase alpha-subunit (EC 1.14.12.10), phenylacetaldehyde dehydrogenase (EC 1.2.1.39), protocatechuate 3,4-dioxygenase (EC 1.13.11.3), butyryl-coenzyme A dehydrogenase (EC 1.3.99.2), aldehyde dehydrogenase (EC 1.2.1.3), and acetyl-coenzyme A (CoA) acetyltransferase (EC 2.3.1.9). Also, the genes for antibiotic resistance to spectinomycin 9-*O*-adenylyltransferase, β-lactamase (EC 3.5.2.6), multidrug resistance transporter, Bcr/CflA family and resistance-nodulation-division (RND) efflux systems, outer membrane lipoprotein, arsenate reductase (EC 1.20.4.1), and cobalt-zinc-cadmium resistance protein (EC 6.3.2.12) were found.

### Nucleotide sequence accession numbers.

The draft genome sequence of *A. baumannii* IITR88 is available in DDBJ/EMBL/GenBank under the accession no. LQAR00000000. The version described in this paper is the first version, LQAR01000000.
