# Correction for Regar et al., “Draft Genome Sequence of Acinetobacter baumannii IITR88, a Bacterium Degrading Indoles and Other Aromatic Compounds”

**DOI:** 10.1128/MRA.01093-18

**Published:** 2018-08-30

**Authors:** Raj Kumar Regar, Vivek Kumar Gaur, Gayatri Mishra, Sudhir Jadhao, Mohan Kamthan, Natesan Manickam

**Affiliations:** a Environmental Biotechnology Laboratory, Environmental Toxicology Group, CSIR-Indian Institute of Toxicology Research (IITR), Vishvigyan Bhawan, Lucknow, India; b Bionivid Technology Pvt. Ltd., Bengaluru, Karnataka, India; c Department of Biochemistry, School of Dental Sciences, Babu Banarsi Das University, Lucknow, India

## AUTHOR CORRECTION

Volume 4, no. 2, e00065-16, 2016, https://doi.org/10.1128/genomeA.00065-16. Page 1: The author affiliations should read as given in this correction.

